# Bilateral Choanal Atresia in a 42-year-old Patient: A Rare Condition Case Report

**DOI:** 10.1007/s12070-024-04491-4

**Published:** 2024-01-23

**Authors:** Sávio Luquetti Silva Vieira, Cecy de Fátima Amiti Fabri, Gabriela Cardoso Lima, Marina Bandoli de Oliveira Tinoco, Maria Elena Padín-Iruegas, Paulo Tinoco, França Vieira e Silva

**Affiliations:** 1 Department of Medicine, Nova Iguaçu University, Rio de Janeiro, 28300000 Brazil; 2 Human Anatomy Area, University of Vigo, Lagoas-Marcosende, s/n, Vigo, 36310 Spain; 3 Department of Medicine and Dentistry, University of Santiago de Compostela, Santiago de Compostela, 15704 Spain

**Keywords:** Choanal atresia, Choanal imperforation, Endoscopic nasal surgery, Rhinorrhea, Charge syndrome

## Abstract

Choanal atresia is an uncommon condition with an incidence of 1:5,000–8,000 live births, affecting females more frequently and often associated with other malformations. This case report presents a 42-year-old female patient who was born with bilateral choanal atresia and intervened surgically for the first time at birth. However, the formed orifice was reobstructed a few months afterward, necessitating reoperation in adulthood. The purpose of this case report is to describe bilateral choanal atresia in detail, including its clinical presentation, epidemiology, diagnosis, pathogenesis, and therapeutic approach. It aims to enhance understanding of this rare but significant condition.

## Introduction

Choanal atresia, characterized by the complete blockage of the choanae, is a rare congenital malformation of the nasal cavity. It can manifest unilaterally (affecting one nostril) or bilaterally (affecting both nostrils). When unilateral, it may present with mild or no symptoms, such as unilateral nasal discharge when lying down and difficulty swallowing. However, it is not life-threatening. Moreover, unilateral choanal atresia is more common for the obliteration to be on the right side [1].

In cases where the blockage of the choanae is bilateral, it poses a life-threatening condition with prominent symptoms, including dyspnea (difficulty breathing, often accompanied by noisy breathing), intermittent cyanosis (bluish discoloration of the skin, particularly during crying or when the mouth is closed), and respiratory failure (severe impairment of breathing, potentially leading to life-threatening complications). Prompt recognition and appropriate treatment are crucial for managing bilateral choanal atresia. Despite its rarity, it is essential to be aware of its clinical manifestations to ensure timely diagnosis and intervention [2].

Choanal atresia diagnosis involves a combination of clinical evaluation, history, and complementary tests. Sinus tomography and nasofibroscopy are the primary diagnostic modalities, providing information about the location, extent, and nature of the atretic plate (bone, membranous, or mixed). This information guides surgical planning [3].

Initial treatment focuses on preserving a patent airway, often through oral airway placement or nasal intubation. Once the airway is secure, definitive surgical correction is performed. Currently, there are several techniques used: microscopic transnasal approach, a traditional method of a surgical microscope to visualize the atretic plate and create an opening between the nasal cavity and nasopharynx; endoscopic transnasal technique, a minimally invasive approach that uses an endoscope to guide the surgeon in creating an opening through the nasal cavity; a transpalatal approach, a procedure that establishes access to the choanae from below by creating an opening through the roof of the mouth (palate), what is particularly effective for thick, cartilaginous atretic plates; a transseptal approach, a technique involving an incision in the nasal septum, dividing wall between the nostrils, to reach the choanae, often combined with other approaches; intranasal dilation, a minimally invasive technique that utilizes dilators or other instruments to mechanically widen the atretic plate, typically considered for mild cases [3]. Among these techniques, the transnasal endoscopic route is the most commonly employed approach [2, 3].

For the present study, the transnasal endoscopic approach was chosen as the preferred surgical technique. This choice is supported by its minimal rate of intraoperative and postoperative complications, faster recovery, excellent visualization of the surgical site, reduced traumatic injury to the patient, and more precise surgery, leading to a lower restenosis rate [3]. 

## Case Report

**Fig. 1:**
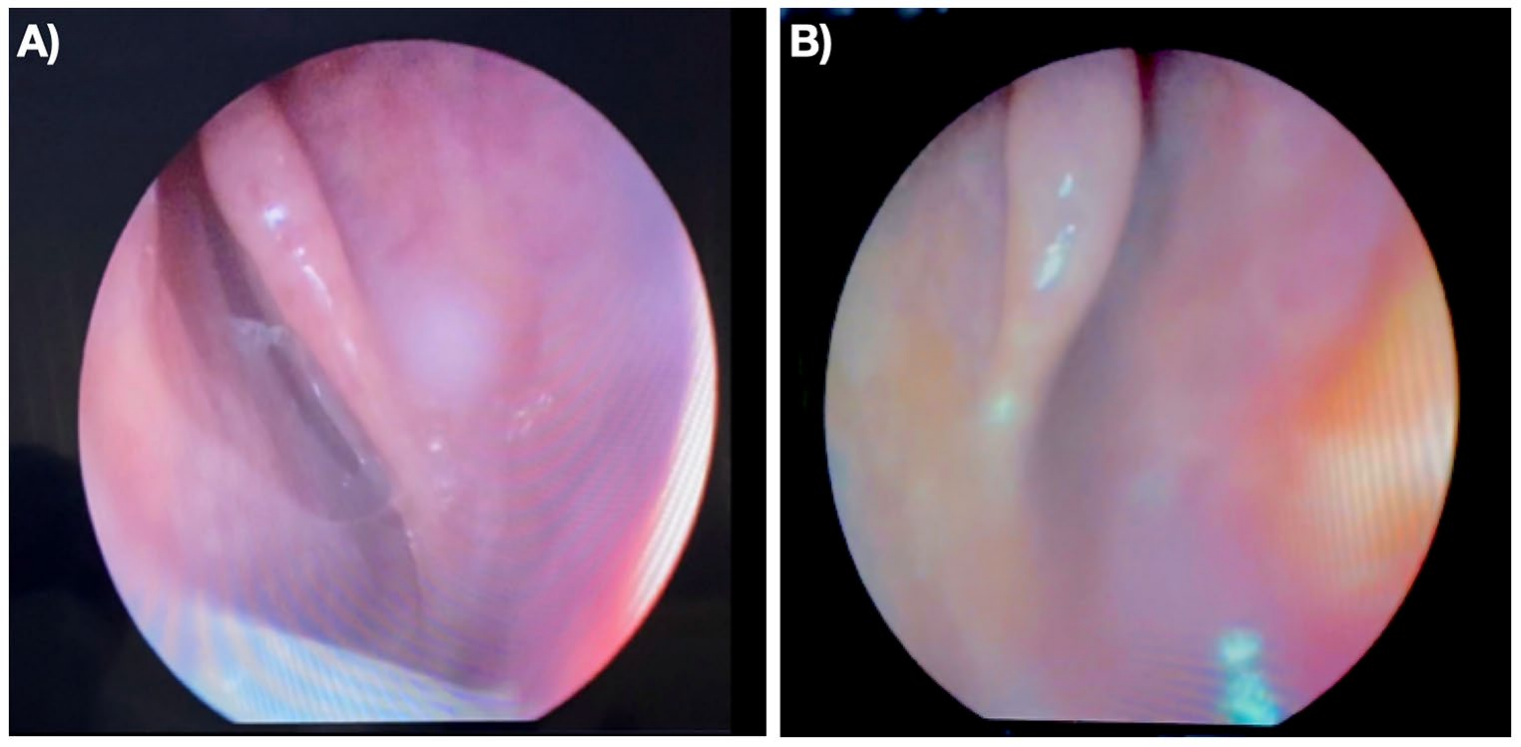
Nasal endoscopy of the right (**A**) and left (**B**) nostrils

**Fig. 2:**
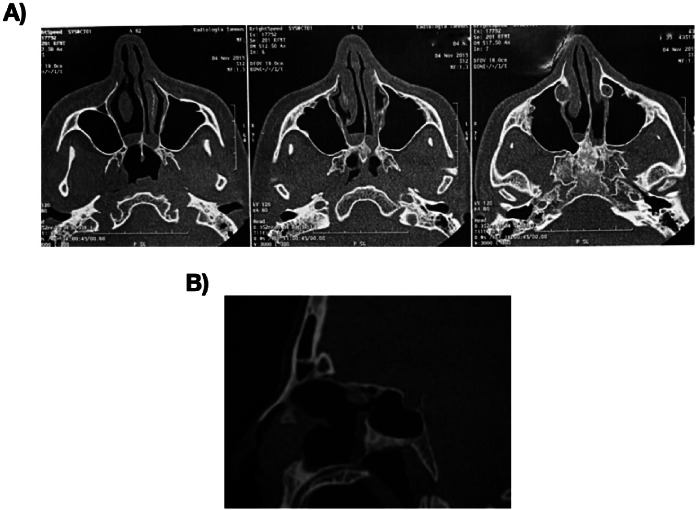
Computed tomography of the facial sinuses showing bilateral osteo-membranous choanal atresia in an axial section (**A**) and sagittal section (**B**)

A 42-year-old female patient, J.A.V., presented to the otorhinolaryngology clinic with persistent nasal obstruction, clear rhinorrhea, and anosmia dating back to childhood. Upon examination, she exhibited an elongated facial structure and exhibited mouth breathing patterns. A nasal endoscopy revealed bilateral occlusion of the posterior choanae (Fig. 1A/B). Computed tomography (CT) imaging of the paranasal sinuses confirmed the diagnosis of bilateral osteo-membranous choanal atresia (Fig. 2A/B).

The patient reported undergoing surgical intervention for bilateral choanal atresia as a newborn, but the openings had reoccluded after the procedure.

Based on the diagnostic findings and clinical history, the patient underwent a two-stage endoscopic nasal surgery. During the first stage, the left posterior choana was opened. Approximately two months later, the right posterior choana was also surgically opened. A follow-up nasal endoscopy confirmed that both choanae were wide open and patent. The patient’s condition improved significantly, and she regained normal nasal breathing.

## Discussion

Choanal atresia occurs in approximately 1 in 8,000 live births. Unilateral cases are more common than bilateral cases, and females are more frequently affected. It can present as an osteo-membranous plate (70% of cases), bony (20%), or membranous (10%) [4].

Several theories seek to explain the etiology of choanal atresia, including the persistence of the buccopharyngeal membrane, excessive proliferation of epithelial cells within the nasal cavities during the 6th to 8th week of gestation, aberrant migration of neural crest cells, or excessive growth of the vertical and horizontal processes of the palatine bones. Furthermore, it is essential to emphasize the anatomical relationship in choanal atresia, where the sphenoid bone forms the superior boundary, the pterygoid plates flank the lateral aspect, the vomer anchors the medial side, and the hard palate serves as the inferior foundation [5].

The main symptoms presented in bilateral choanal atresia, when diagnosed late, are nasal obstruction, hyaline rhinorrhea, and mouth breathing. Nasofibroscopy is available with either a rigid endoscope or flexible fiberoscopy, allowing for the analysis of nasal characteristics and choanal atresia. Computed tomography (CT) imaging is essential, providing a detailed evaluation of atresia characteristics, lamina thickness, vomer thickening, and narrowing of the nasal cavity, facilitating surgical planning [1].

The treatment of choanal atresia should initially be to maintain a patent airway until a definitive approach is made. In this, the objective is to restore nasal airflow, preventing damage to facial growth, using a quick and safe technique. The most commonly used technique currently is endoscopic transnasal, because it is less traumatic, has lower complication rates, demonstrates good visualization, and offers better post-operative recovery [3].

The patient in this case report was approached at the age of 42 using the transnasal endoscopic technique in two stages, with an interval of 2 months between the two approaches. The patient is progressing satisfactorily approximately 6 months after surgery. Reports improvement in symptoms, and nasal breathing, and when nasal endoscopy was performed, there was communication between the nasal cavity and the rhinopharynx. However, it is not yet known how the situation will evolve in the long term.

## Conclusion

The presentation of this case and discussion on the subject is important in otorhinolaryngology practice, for the recognition of this pathology, and also its approach, with emphasis on cases of bilateral choanal atresia, based on correct diagnosis and therapeutic management, an emergency scenario is modified to improve the patient’s quality of life, as in the present study.
